# Dispersion of ventricular repolarization in relation to cardiovascular risk factors in hypertension

**Published:** 2014

**Authors:** A Ciobanu, GS Gheorghe, M Ababei, M Deaconu, AM Ilieşiu, M Bolohan, N Păun, C Nicolae, IT Nanea

**Affiliations:** * “Theodor Burghele” Clinical Hospital, Bucharest, Romania; ** “Carol Davila” University of Medicine and Pharmacy, Bucharest, Romania

**Keywords:** QT interval, Tpeak-Tend interval, cardiovascular risk factors, SCORE model

## Abstract

Rationale: Hypertension associates with sudden cardiac death, its relationship with ventricular arrhythmias being demonstrated by multiple studies, an increased dispersion of repolarization being an important arrhythmogenesis mechanism. Only a small percentage of hypertensive patients presents increased blood pressure values exclusively as risk factor, most of them presenting additional risk factors that reinforce each other leading to increased total cardiovascular risk.

Aim: to analyze the dispersion of repolarization parameters (QT interval, QT dispersion, Tpeak-Tend interval (Tpe), Tpe/QT, Tpe dispersion) in relation to cardiovascular risk factors, as well as total cardiovascular risk estimated by SCORE model, in mild to moderate essential hypertension.

Method: 62 consecutive patients, mean age 55±11 years, were evaluated by 24 hours Holter electrocardiography monitoring. Manual measurement of dispersion of repolarization parameters was performed.

Results: Based on SCORE model, 33.9% patients presented low risk, 40.3% moderate risk, 16.1% high risk and 9.1% very high risk. Age had a positive correlation with QT and reverse correlations with QT dispersion, Tpe and Tpe/QT in lead V1. Tpe/QT showed significantly higher values in men versus women. Glucose metabolism disorders associated with higher values of QT and Tpe dispersion were present. However, dispersion of repolarization parameters was similar between risk categories assessed by SCORE model, Tpe in lead V3 correlated significantly with SCORE score.

Conclusions: In mild to moderate hypertension, the dispersion of repolarization parameters is influenced by age, gender and glucose metabolism disorders. Among these, Tpe in lead V3 correlates with total cardiovascular risk assessed by SCORE model.

**Abbreviations:** dTpe = T peak – T end interval dispersion, ECG = electrocardiography, Tpe = T peak - T end interval, Tpe/QT = T peak – T end / QT ratio, QTd = QT dispersion

## Introduction

Cardiovascular diseases account for one third of the deaths worldwide, with a growing contribution to the global burden of the disease [**1**]. Of these, hypertension has an increasing prevalence, mainly due to the increasing longevity and prevalence of behavioral contributors factors such as obesity, physical inactivity and unbalanced diet [**2**,**3**].

Hypertension associates with sudden cardiac death, its relationship with ventricular arrhythmias being demonstrated by multiple studies [**1**]. Several mechanisms were proposed in order to explain this association, involving both structural and electrophysiological myocardial changes. The electrical ventricular remodeling in hypertension includes nonuniform prolongation of action potential and heterogeneity of the duration of refractory periods and the conduction velocities of adjacent myocardial areas. All these changes are referred to as increased dispersion of ventricular repolarization, a concept which fundaments on the electrophysiological distinct features of three different ventricular cell types - epicardial, endocardial and mid-myocardial (M cells) [**2**]. Normally, action potentials are shorter in epicardial than in endocardial cells, while M cells have the longest action potentials. These differences contribute substantially to the morphology and duration of the electrocardiographic T wave [**4**]. Subsequently, several non-invasive electrocardiographic (ECG) parameters were proposed for the quantification of the dispersion of repolarization such as QT interval duration, QT dispersion (QTd), T wave micro alternans, and more recently, T peak – T end interval (Tpe), T peak - T end/QT ratio (Tpe/QT) and T peak – T end interval dispersion (dTpe). Experimental studies have shown that the peak of the T wave coincides with the full repolarization of the epicardial cells and the end of the T wave with the full repolarization of the M cells [**5**], so the Tpe interval is an index of dispersion of ventricular repolarization [**4**,**6**,**7**], being useful to stratify the arrhythmic risk [**5**,**8**-**10**].

Starting from the observation that only a small percentage of hypertensive patients presents as a risk factor increased blood pressure values exclusively, while most of them present additional risk factors that reinforce each other, a system of total cardiovascular risk assessment was decided to be implemented to maximize the cost/efficiency ratio in the management of hypertension [**11**]. Of the many computerized methods developed for total cardiovascular risk estimation [**12**-**16**], the European Society of Hypertension recommends the SCORE (Systematic Coronary Risk Evaluation) model. This score estimates risk of fatal cardiovascular events in 10 years, taking into account age, gender, smoking status, total cholesterol and systolic blood pressure values [**16**].

In this light, the purpose of this study was to analyze the parameters of dispersion of repolarization (QT, QTd, Tpe, Tpe/QT and dTpe) in relation to unmodifiable (age, gender) and modifiable (smoking, obesity, dyslipidemia, glucose metabolism disorders) cardiovascular risk factors, as well as total cardiovascular risk estimated by the SCORE model, in patients with mild to moderate essential hypertension.

## Method

62 consecutive patients with grades I and II essential hypertension diagnosed according to the European Guidelines for the Management of Hypertension [**11**], in sinus rhythm, were included. Exclusion criteria were: secondary hypertension, documented ischemic heart disease (positive ECG stress test, coronary angiography with significant stenosis, prior myocardial infarction), heart failure, severe valvular disease, atrial fibrillation, bundle branch blocks, pre-excitation syndromes, chronic kidney disease stages III-V, electrolyte disturbances, treatment with antiarrhythmic drugs.

Based on the SCORE score and the presence of cardiovascular risk factors, the following risk categories were defined: low risk (SCORE <1%), moderate risk (SCORE ≥1% and <5%), high risk (markedly elevated single risk factor or SCORE ≥5% and <10%) and very high risk (presence of diabetes mellitus or SCORE ≥10%) [**11**].

The patients were evaluated by 24 hours Holter ECG monitoring, using a true 12 lead continuous recording device. A manual measurement of the dispersion of repolarization parameters was performed on its traces. The QT interval was defined as the interval between the onset of the QRS complex and the end of the T wave and was measured in all leads. Its maximal value was taken into account. Tpe was defined as the interval between the peak and end of the T wave and was measured in the precordial leads. The mean and maximal values of this interval have also been taken into account. The end of the T wave was measured for both Tpe and QT intervals, by convention and by using the method of the tangent to the steepest slope of the descending portion of the T wave. 3 consecutive measurements in the same lead were performed for each interval and then the arithmetic mean was calculated. QTd and dTpe were defined as the difference between the highest and lowest value of QT and Tpe intervals, respectively. QT interval, QTd, Tpe and dTpe were corrected to heart rate by using Bazett formula.

Data collection was performed by using a database created with Microsoft Access 2010, respecting the confidentiality of the patients.

Statistical analysis:

The results were presented as mean ± standard deviation for numeric variables and as absolute numbers and percentages for categorical variables. For the analysis of numeric variables, parametric (Student's t-test, ANOVA; for normally distributed data) or non-parametric (Mann-Whitney, Kruskal-Wallis; for data without a normal distribution) tests were used. Linear regression and Pearson correlation coefficient r (continuous numerical variables with normal distribution) or Spearman test with Spearman’s rank correlation coefficient ρ (continuous numeric variables without normal distribution or correlations between ordinal variables and continuous numeric variables) were used for correlations between numerical variables. The statistical significance was considered for a p-value <0.05. The statistical analysis was performed by using STATISTICA 8.0.

## Results

The basic characteristics of the subjects are listed in **Table 1**.

**Table 1:** Basic characteristics of the analyzed group

**Parameters**	**Total (n=62)**
**Age (years)**	55 ± 11
**Male gender**	26 (41.9%)
**Body mass index (kg/m2)**	29.2 ± 4.8
Left ventricular mass index* (g/m2)	92.10 ± 20.77
**Risk factors**	
**Smoking**	12 (19.3%)
**Dyslipidemia**	33 (53.2%)
**Intermediate hyperglycemia**	24 (38.7%)
**Diabetes**	6 (9.6%)
**Obesity**	29 (46.7%)
**Total cardiovascular risk assessment using SCORE model**	
**SCORE score**	3.0 ± 2.5
**Low additional risk**	21 (33.9%)
**Medium additional risk**	25 (40.3%)
**High additional risk**	10 (16.1%)
**Very high additional risk**	6 (9.7%)
*calculated according to Devreux formula

Age had a positive correlation with QT (r = 0.27, p = 0.02) and reverse correlations with QTd (r = -0.31, p = 0.01) and Tpe in lead V1 (r = -0.37, p = 0.03). The correlations of dispersion of repolarization parameters with age are depicted in **Table 2**.

**Table 2:** Correlations of parameters of dispersion of repolarization with age

**Parameters**	**Age**	
	**r**	**p**
**QT**	**0.27**	**0.02**
**QTd**	**-0.31**	**0.01**
**Tpe V1**	**-0.37**	**0.03**
**Tpe/QT V1**	-0.33	0.05
**Tpe V2**	0.05	0.67
**Tpe/QT V2**	-0.08	0.53
**Tpe V3**	0.06	0.63
**Tpe/QT V3**	-0.01	0.89
**Tpe V4**	-0.04	0.71
**Tpe/QT V4**	-0.15	0.24
**Tpe V5**	-0.06	0.62
**Tpe/QT V5**	-0.16	0.22
**Tpe V6**	-0.04	0.75
**Tpe/QT V6**	-0.15	0.22
**Mean Tpe**	-0.06	0.63
**Mean Tpe/QT **	-0.17	0.17
**Maximum Tpe **	-0.01	0.89
**Maximum Tpe/QT **	-0.10	0.41
**dTpe**	0.11	0.35
*r – Pearson correlation coefficient		

Only Tpe/QT showed significant higher values in men versus women (**Table 3**) with respect to gender.

**Table 3:** Mean values of dispersion of repolarization parameters according to gender

**Parameters**	**Women (n=36;58.1%)**	**Men (n=26; 41.9%)**	**p**
**QT (ms)**	424.2 ± 25.7	417.5 ± 265.9	0.32
**QTd (ms)**	46.2 ± 19.7	49.0 ± 16.0	0.54
**Tpe V1 (ms)**	56.0 ± 11.3	63.8 ± 12.2	0.06
**Tpe/QT V1**	**0.1409 ± 0.0262**	**0.1695 ± 0.0348**	**0.009**
**Tpe V2 (ms)**	70.0 ± 13.0	76.3 ± 12.1	0.05
**Tpe/QT V2**	**0.1766 ± 0.0294**	**0.1922 ± 0.0286**	**0.04**
**Tpe V3 (ms)**	72.0 ± 16.4	75.7 ± 10.7	0.30
**Tpe/QT V3**	0.1762 ± 0.0357	0.1898 ± 0.0273	0.10
**Tpe V4 (ms)**	67.9 ± 14.2	72.9 ± 10.7	0.13
**Tpe/QT V4**	0.1677 ± 0.0334	0.1832 ± 0.0271	0.05
**Tpe V5 (ms)**	66.8 ± 12.5	69.1 ± 11.7	0.47
**Tpe/QT V5**	0.1630 ± 0.0271	0.1716 ± 0.0282	0.24
**Tpe V6 (ms)**	61.1 ± 10.3	64.9 ± 12.3	0.20
**Tpe/QT V6**	**0.1474 ± 0.0273**	**0.1612 ± 0.0273**	**0.03**
**Mean Tpe (ms)**	66.2 ± 9.9	70.9 ± 8.0	0.05
**Mean Tpe/QT **	**0.1636 ± 0.0225**	**0.1786 ± 0.0204**	**0.009**
**Maximum Tpe (ms)**	78.4 ± 13.5	81.6 ± 10.2	0.31
**Maximum Tpe/QT **	**0.1915 ± 0.0277**	**0.2056 ± 0.0246**	**0.04**
**dTpe (ms)**	25.0 ± 12.7	22.7 ± 9.2	0.42

Taking into account the modifiable cardiovascular risk factors, the presence of glucose metabolism disorders (which include impaired fasting glucose, impaired glucose tolerance and diabetes mellitus) associated with higher values of QT (428,3 ± 28,2 ms versus 415,0 ± 21,9 ms, p = 0,04) and dTpe (27,6 ± 12,9 ms versus 20,7 ± 8,5 ms, p = 0,01) compared to normoglycemia.

Neither smoking status, nor obesity or dyslipidemia had influences on the parameters of dispersion of repolarization.

Though we have not found any difference between the additional risk categories assessed by SCORE model in the parameters of dispersion of repolarization (**Table 4**), Tpe in lead V3 correlated significantly with SCORE score (Spearman’s rank correlation coefficient ρ = 0.26, p = 0.04)

**Table 4:** Mean values of dispersion of repolarization parameters according to SCORE risk categories

**Parameters**	**Low risk (n=21;33,9% )**	**Moderate risk (n=25;40,3%)**	**High risk (n=10;16,1%)**	**Very high risk (n=6; 9,7%)**	**p**
**QT (ms)**	417.3±26.1	418.6±25.5	435.1±28.5	424.3±18.4	0.30
**QTd (ms)**	48.2±17.9	44.9±12.7	49.9±29.1	50.1±20.0	0.90
**Tpe V1 (ms)**	59.5±16.5	57.7±10.2	61.4±11.0	58.2±9.4	0.94
**Tpe/QT V1**	0.1490±0.0410	0.1518±0.0313	0.1553±0.0232	0.1523±0.0306	0.98
**Tpe V2 (ms)**	71.0±13.0	71.9±13.2	72.6±6.7	81.2±18.4	0.39
**Tpe/QT V2**	0.1813±0.0302	0.1843±0.0305	0.1790±0.0239	0.1924±0.0398	0.83
**Tpe V3 (ms)**	68.8±14.1	74.6±14.9	75.3±9.5	82.9±16.1	0.16
**Tpe/QT V3**	0.1716±0.0355	0.1865±0.0322	0.1818±0.0206	0.1989±0.0394	0.25
**Tpe V4 (ms)**	66.6±12.1	72.9±13.7	67.5±11.0	75.7±14.3	0.25
**Tpe/QT V4**	0.1685±0.0326	0.1828±0.0315	0.1625±0.0268	0.1825±0.0319	0.24
**Tpe V5 (ms)**	66.5±13.6	68.2±11.0	63.7±8.2	76.4±14.9	0.23
**Tpe/QT V5**	0.1636±0.0323	0.1695±0.0196	0.1521±0.0183	0.1852±0.0416	0.10
**Tpe V6 (ms)**	61.8±12.3	62.1±9.0	63.7±14.3	67.1±12.6	0.76
**Tpe/QT V6**	0.1520±0.0295	0.1537±0.0176	0.1496±0.0321	0.1620±0.0276	0.71
**Mean Tpe (ms)**	66.2±9.8	68.7±9.6	68.0±5.3	73.4±12.1	0.43
**Mean Tpe/QT **	0.1657±0.0258	0.1733±0.0210	0.1643±0.0125	0.1789±0.0308	0.43
**Maximum Tpe (ms)**	78.5±11.5	79.4±13.7	80.1±7.6	84.9±15.9	0.74
**Maximum Tpe/QT **	0.1947±0.0280	0.1989±0.0280	0.1936±0.0147	0.2064±0.0390	0.77
**dTpe (ms)**	23.8±13.2	23.3±10.9	23.2±7.5	29.3±12.4	0.70

## Discussions

Data from studies on the relationship between QT and QTd and age are contradictory, probably because of different methodologies (measurement method, heart rate correction, the presence of additional risk factors and comorbidities that could be confounders). A recent study conducted on a group of 198 healthy subjects reported a statistically significant correlation between QT and age, even after the adjustment for factors such as smoking status, body mass index, blood pressure values [**17**]. The same study suggests the absence of a correlation between QTd and age [**17**]. However, a more extensive study, involving 1,096 healthy subjects, reported values of QTd progressively smaller with age [**18**]. Data on variations according to age of the new parameters of dispersion of repolarization are insufficient.

The present study revealed a significant correlation of QT and QTd according to age (r = 0.27, p = 0.02, r = 0.31, p = 0.01, respectively). Among the new parameters only repolarization Tpe in lead V1 correlated reversely according to age (r = -0.37, p = 0.03).

It has been shown that the duration of the QT interval is greater in young women than in men, a difference that becomes increasingly smaller in the 5th decade and disappears in older age [**19**,**20**]. QT shortening effect in males appears in adolescence and is considered to be due to the secretion of testosterone [**19**,**21**,**22**].

Regarding QTd, some studies report no significant gender differences [**23**] and other that higher values are found in men [**18**,**24**].

The data are controversial on Tpe interval [**25**]. Analyzing a sample aged between 0 and 88 years, Nakagawa et al. noted that for the age group between 30 and 70 years, Tpe in lead V5 has significantly lower values in women versus men and its values are similar between sexes for the remaining age groups [**26**]. Moreover, on a sample of 40 subjects between 35 and 67 years, assessing Tpe in leads V3-V6, Mayuge et al. identified significantly lower values of this parameter in women compared to men [**27**]. In contrast, data published by Smetana et al. reported higher values of Tpe in women [**28**].

Our study revealed significantly higher values in men of Tpe/QT in leads V1, V2, V6, and mean and maximum Tpe/QT, with borderline significance for Tpe. The research reported so far, suggests that between these two parameters, Tpe/QT would be a more sensitive index of arrhythmogenesis, estimating repolarization dispersion relative to the entire duration of repolarization and eliminating confounding factors such as changes in heart rate and inter-individual variations of QT interval [**29**].

There are also contradictions regarding the effects of smoking on QT and QTd. While Mestre et al. did not identify any effects of the smoking status on QT [**30**], Ileri et al. reported higher values for QT and QTd in smokers [**31**] and Dilaveris et al. observed significantly lower values of QT in smokers, without differences in QTd [**32**]. Two of the most recent studies also assessing the new parameters of dispersion of repolarization, showed significantly higher values of QT, QTd, Tpe and Tpe/QT in smokers [**33**,**34**]. Our study identified similar values of all parameters between smokers and nonsmokers.

There are studies reporting elevated QT in obese patients, the explanation given being related to changes in sympathetic-parasympathetic balance of autonomic tone [**35**-**37**], but the relationship between QTd and obesity is controversial [**38**]. A recent study also comparing the new parameters of dispersion of repolarization between subjects with obesity without other comorbidities and normal weight subjects, reported no statistically significant differences between the two groups in QT values [**35**]. Neither the present study identified any effect of obesity on repolarization dispersion parameters.

The relationship between dyslipidemia and dispersion of ventricular repolarization did not benefit from too many research reported in literature. A recent case-control study including 40 dyslipidemic patients identified significantly higher values of QT in these patients versus controls [**39**]. The rest of the repolarization dispersion parameters were not sufficiently evaluated in relation to dyslipidemia. Our study did not identify an association of dyslipidemia with changes in the dispersion of ventricular repolarization.

Most of the studies of patients with diabetes report QT prolongation compared with non-diabetic patients [**37**,**40**,**41**]. There are also studies suggesting that 30% of the obese patients with intermediate hyperglycemia have higher values of QT [**42**]. A very recent case-control study identifies higher values of QTd, Tpe and Tpe/QT in lead V5 in patients with type 2 diabetes compared to healthy subjects, with similar values of QT between these groups [**43**]. The present study, analyzing repolarization dispersion parameters in relation to the presence of glucose metabolism disorders (including intermediate hyperglycemia and diabetes) showed QT and dTpe prolongation in these patients compared to normoglycemic patients.

Regarding the relationship between parameters of dispersion of repolarization and total cardiovascular risk in hypertensive patients, literature data is scarce. A recent study including 48 patients with hypertension, mean age 59.4 years, with 54.2% patients at high and very high risk estimated by the SCORE model, reported an association of QT and Tpe with the level of total cardiovascular risk [**44**]. There were no statistically significant differences in values of dispersion of repolarization parameters between risk categories assessed by SCORE model in our study. Tpe in lead V3 was the only parameter that correlated with SCORE score (ρ = 0.26, p = 0.04). We consider these results in the context of the characteristics of our study group, with younger patients (mean age 55 years) and only 25.8% of them being at high or very high additional risk estimated by SCORE model. Thus, from all parameters exploring dispersion of ventricular repolarization, Tpe in lead V3 probably has a higher sensitivity for the highlighting of the relationship between electrophysiological changes and total cardiovascular risk.

## Conclusions

In patients with mild to moderate hypertension, parameters of dispersion of ventricular repolarization are influenced according to age, gender and presence of glucose metabolism disorders. Among these, Tpe in lead V3 is being remarked, correlating with total cardiovascular risk assessed by SCORE model. Smoking, obesity and dyslipidemia do not appear to influence myocardial repolarization.

**Acknowledgement:** This paper is supported by the Sectoral Operational Programme Human Resources Development (SOP HRD) 2007-2013, financed from the European Social Fund and by the Romanian Government under the contract number POSDRU/107/1.5/S/82839.

**Disclosures:** None.
